# Supplementary material for: Polyketide synthase gene clusters in the ericoid mycorrhizal fungus Oidiodendron maius: genome-wide prediction, expression profiling, and functional characterization of knockout mutants
Source: Front Plant Sci. 2026 Sep 16;17:1924430. doi: 10.3389/fpls.2026.1924430 (PMC13623903; doi:10.3389/fpls.2026.1924430)
Supplement: Supplementary file 1 [file SupplementaryFile1.zip › Supplementary Materials/Supplementary Folder 1.zip!/Oidma1_AssemblyScaffolds_Repeatmasked/knownclusterblast/cluster50/jgi.p_Oidma1_106840_mibig_hits.html]

| MIBiG Protein | Description | MIBiG Cluster | MiBiG Product | % ID | % Coverage | BLAST Score | E-value |
| --- | --- | --- | --- | --- | --- | --- | --- |
| ACR44939.1 | 8-amino-7-oxononanoate\_synthase | BGC0001238 | Biotin\_biosynthetic\_gene\_cluster | 36 | 83.0107526882 | 245 | 9.3e-65 |
| ACR44942.1 | 8-amino-7-oxononanoate\_synthase | BGC0001239 | Biotin\_biosynthetic\_gene\_cluster | 36 | 83.0107526882 | 245 | 9.3e-65 |
| CAA16183.1 | polyketide\_synthase | BGC0001063 | Undecylprodigiosin\_biosynthetic\_gene\_cluster | 26 | 80.0 | 124 | 2.4e-28 |
